# Extraction and separation of astaxanthin with the help of pre-treatment of *Haematococcus pluvialis* microalgae biomass using aqueous two-phase systems based on deep eutectic solvents

**DOI:** 10.1038/s41598-024-55630-4

**Published:** 2024-03-05

**Authors:** Neda Nemani, Seyed Mohsen Dehnavi, Gholamreza Pazuki

**Affiliations:** 1 Department of Chemical Engineering, Amirkabir University of Technology (Tehran Polytechnic), Tehran, Iran; 2 Department of Cell and Molecular Biology, Faculty of Life Science and Biotechnology, Shahid Beheshti University, P.O. Box 1983969411, Tehran, Iran

**Keywords:** *Haematococcus pluvialis* microalgae, Astaxanthin, Aqueous two-phase system, Deep eutectic solvent, Distribution coefficient, Pre-treatment, Chemical engineering, Biotechnology, Engineering

## Abstract

The microalgae *Haematococcus pluvialis* are the main source of the natural antioxidant astaxanthin. However, the effective extraction of astaxanthin from these microalgae remains a significant challenge due to the rigid, non-hydrolyzable cell walls. Energy savings and high-efficiency cell disruption are essential steps in the recovery of the antioxidant astaxanthin from the cysts of *H. pluvialis*. In the present study, *H. pluvialis* microalgae were first cultured in Bold's Basal medium under certain conditions to reach the maximum biomass concentration, and then light shock was applied for astaxanthin accumulation. The cells were initially green and oval, with two flagella. As the induction time increases, the motile cells lose their flagellum and become red cysts with thick cell walls. Pre-treatment of aqueous two-phase systems based on deep eutectic solvents was used to decompose the cell wall. These systems included dipotassium hydrogen phosphate salt, water, and two types of deep eutectic solvents (choline chloride–urea and choline chloride–glucose). The results of pre-treatment of *Haematococcus* cells by the studied systems showed that intact, healthy cysts were significantly ruptured, disrupted, and facilitated the release of cytoplasmic components, thus facilitating the subsequent separation of astaxanthin by liquid–liquid extraction. The system containing the deep eutectic solvent of choline chloride–urea was the most effective system for cell wall degradation, which resulted in the highest ability to extract astaxanthin. More than 99% of astaxanthin was extracted from *Haematococcus* under mild conditions (35% deep eutectic solvent, 30% dipotassium hydrogen phosphate at 50 °C, pH = 7.5, followed by liquid–liquid extraction at 25 °C). The present study shows that the pre-treatment of two-phase systems based on deep eutectic solvent and, thus, liquid–liquid extraction is an efficient and environmentally friendly process to improve astaxanthin from the microalgae *H. pluvialis*.

## Introduction

Microalgae are a diverse group of single-celled photosynthetic species with a microscopic structure that converts sunlight into chemical energy using a carbon source. To date, more than 30,000 species of microalgae have been identified that grow in freshwater and marine waters^1^. These microorganisms are considered very promising for the production of multifunctional biomass because they can proliferate, produce large amounts of lipids^2^, carbohydrates, and proteins, and are the source of biomolecules in the cosmetics and pharmaceutical industries^3,4^. They are also widely used in the aquaculture and poultry industries^2^.

*Haematococcus pluvialis* is a freshwater bivalve single-celled microalgae of the genus Chlorophyceae, order Volvocales, the family *Haematococcus* aureus, and the species *H. pluvialis*^5,6^. This microalga can produce resistant cells called cysts or aplanospores under stress^7^, which causes the accumulation of astaxanthin and fat in the cell. Astaxanthin (3,3'-dihydroxy-β, β-carotene-4,4'-dione) is a secondary carotenoid belonging to the xanthophyll group that is widely used as a source of fat-soluble pigments, nutritional supplements, antioxidants, and anti-cancer drugs^8–10^.

To produce astaxanthin from microalgae, three essential steps, including cultivation, harvesting, and extraction, must be taken. From these three stages, the extraction of astaxanthin is considered the main challenge because the stress conditions that cause the accumulation of astaxanthin in microalgae cause the formation of rigid cell walls, increase the mechanical and chemical resistance of microalgae cells, and prevent solvent access to the cells. The cell wall of *H. pluvialis* is very resistant to cell disruption, such as mechanical and chemical pre-treatment, and the extraction of astaxanthin may be complicated. Therefore, disrupting the cell wall is an effective process to improve the extraction of astaxanthin from healthy cysts^11^.

Physical destruction of the cell wall of *H. pluvialis* has been done by various methods, such as bead milling, high-pressure homogenization, ultrasonication, pulsed electric field pre-treatment, and using supercritical fluid. These methods have advantages such as high efficiency, reliability, and industrial scalability, but they consume a lot of energy and require expensive equipment that is not suitable for industry. Conversely, chemical methods that require organic solvents, acids, and nanomaterials can effectively save energy and are simple. However, the chemicals used must be carefully managed due to their biotoxicity and are not preferred for the production of feed and food from algal crops. Biological methods such as enzyme pre-treatment, germination, and milking have been investigated, which are more compatible with the environment than physical and chemical methods, however, due to their relatively low efficiency and challenges related to the biochemical engineering of the process. need more research^12,13^.

In response to growing environmental concerns, a number of novel methods for astaxanthin pre-treatment and extraction employing ionic liquids have been developed. For the first time in 2018, Zhi et al. looked into the potential of ionic liquids as a brand-new pre-treatment method to damage the healthy *H. pluvialis* cyst cells' cell walls and make astaxanthin extraction easier. After pre-treatment, it produced holes and cavities on the surface of the *Haematococcus* cells, making it easier to extract the astaxanthin by using methanol. This allowed for an extraction of astaxanthin of up to 85.42%^14^. Additionally, Praveen Kumar and associates (2015) looked at a novel approach using short-term germination based on the normal life cycle of *H. pluvialis* as an energy-efficient pre-treatment for the extraction of astaxanthin using ionic liquids. The cyst cell wall was damaged and destroyed during germination, making it easier for IL to extract astaxanthin at room temperature. The yield of astaxanthin above 19.5 pg/cell was produced with this natural pre-treatment using 1-ethyl-3-methylimidazolium ethyl sulfate for a very brief reaction period of 1 min^15^. However, extracting astaxanthin from *Haematococcus* microalgae using the standard ionic liquid extraction method is difficult, expensive, and hazardous^11^. By raising the biological scale of bioseparation while maintaining high purification performance and high product yield, an effective and economical approach must be used. Aqueous two-phase systems (ATPS), which are straightforward, environmentally friendly, easy to scale, and quick to complete, have been suggested as an alternative technique in the separation of biomolecules in recent years. Additionally, the system contains water, which doesn't affect the pH and is inert to biological materials. Studies have revealed that biological materials structures are stable in these systems^16,17^.

In order to address some of the shortcomings of ionic liquids, deMara et al. (2011^18^) proposed deep eutectic solvents (DES), a unique class of green solvents. The two primary elements of DESs are a hydrogen bond acceptor (HBA) like choline chloride or choline acetate and a hydrogen bond donor (HBD) like amides, sugars, amines, alcohols, and carboxylic acids^18,19^. DES has various benefits over traditional ionic liquids, including ease of manufacture, low cost, minimal toxicity, and great biodegradability^17^. In order to separate and enrich chlorogenic acid in blueberry leaves, a natural deep eutectic solvent-based aqueous two-phase system (NADES-ATPS) was originally employed. Selective DNA isolation has been demonstrated to be efficient and ecologically friendly when using DES-ATPS^20^. Additionally, DES-ATPS, which opens up new avenues for protein separation, greatly enhances bovine serum albumin (BSA). In a different study, NADES-ATPS was created as a technique for synthesizing C-PC from *Spirulina platensis* that is eco-friendly, practical, recyclable, and effective^20^.

According to reports, cellulose and hemicellulose make up the majority of the cell walls of *H. pluvialis*. As a result, several DESs are anticipated to efficiently breakdown cellulose by rupturing the hydrogen bond that makes up the main cell wall of cell biomass and microalgae^18–20^. An aqueous two-phase system based on deep eutectic solvents was therefore employed in this work as an eco-friendly means of resolving these problems. In this study, two novel compounds based on choline chloride combined with urea and glucose were employed to create DES based on choline chloride for the breakdown of the cell wall of *Haematococcus* microalgae. This approach uses fresh *H. pluvialis* biomass, which lowers total energy consumption and speeds up the extraction process by avoiding the need to dry and grind up the microalgae cells.

## Method

### Chemicals

Materials required for Bold's Basal medium (BBM) culture medium and organic and inorganic solvents (choline chloride, urea, glucose, dipotassium hydrogen phosphate, 2-propanol, ammonium disulfate, methanol, potassium hydroxide, sodium hydroxide, and 99% hydrochloric acid) German Merck purchased. Standard astaxanthin was also purchased from Sigma Aldrich**.**

### Microalgae strain and culture conditions

The microalgae strain *Haematococcus pluvialis* CCAP 7.34, created by Guilan Aquaculture Research Institute, was utilized in the current investigation. Additionally, culture medium (BBM), whose components are listed in Table S1, was employed^21^.

### Two-stage culture conditions of *H. pluvialis*

For the first stage of culture, 950 ml of fresh BBM culture medium autoclaved for 20 min at 121°C was added to a 1 L Erlenmeyer flask with 50 ml of vegetative green strain *H. pluvialis* CCAP 34.7. To provide the light needed for photosynthesis, a red LED lamp with an output power of 9 watts was used. The intensity of light emitted on Erlenmeyer flasks was measured by a lux meter and set to 3000 lx. The temperature of the microalgae culture medium was set at 25 ± 1°C and pH = 7, and the growth period of the green vegetative stage of *H. pluvialis* microalgae was 14 days to reach the maximum amount of biomass^4,19,22^.

For the cultivation of the second phase, the red phase, a shock or stress must be applied to the microalgae to accumulate astaxanthin. In this research, a light shock was used. Thus, after 14 days, instead of the LED lamp with red light, which had a lower intensity, a moonlight lamp with white light with an output power of 18 watts was used, and the light intensity was set to 7000 lx with a lux meter. Microscopic examination showed changes in cell morphology during the induction period. Motile cells were initially green and oval or pear-shaped with two flagella. As the induction time increased, the motile cells lost their flagella and became red cysts with thick cell walls. In the early induction stage, red pigments caused by astaxanthin accumulation appeared towards the center of green nonmotile cells. The red pigment gradually occupied the entire cell volume, resulting in large red cysts^23^.

### Aqueous two-phase systems based on deep eutectic solvent

#### Synthesis of deep eutectic solvents

In this study, two deep eutectic solvents were synthesized and used to destroy the cell wall of *H. pluvialis*. Deep eutectic solvents show different physical properties according to the substance used in their synthesis and the considered molar ratio. Therefore, according to the characteristics and nature of these solvents, solvents that have the ability to destroy the cell wall of microalgae and are also liquid at ambient temperature should be selected. According to these limitations, the nature of the material, trial and error, and physical properties of deep eutectic solvent materials consisting of choline chloride–urea with a molar ratio of 1:2 and deep eutectic solvent consisting of choline chloride–glucose with a molar ratio of 2:1 are determined.

Choline chloride is very absorbent of moisture, and the existing moisture should be removed completely before use. For this purpose, choline chloride was placed in an oven at 60°C for 24 h to dry the existing moisture. The ingredients of the deep eutectic solvent were measured according to the molar ratio and their weight and poured into a glass vial with a lid. They were heated for a period of 240 min at a temperature of 80 °C on a magnetic–thermal stirrer to create a clear liquid. When all the materials in the container turn into a clear liquid, the solution is finished^20^.

Since choline chloride is highly absorbent of moisture and may absorb some moisture during the preparation of the solvent, after making the deep eutectic solvent, a Karl Fischer titrator was used to measure the water content in the samples. Karl Fischer generally analyzes the amount of remaining water in the material. This method includes iodine that dissolves very well in water, so it is possible to understand the amount of water by displaying the electrical potential of excess iodine. To measure the water content in the samples, the analysis was done by a Karl Fischer titration device (684 KF Coulometer) at the Iranian Institute of Chemistry and Chemical Engineering. The results of the analysis are reported in Table S2.

#### Binodal curve

**Figure 1:**
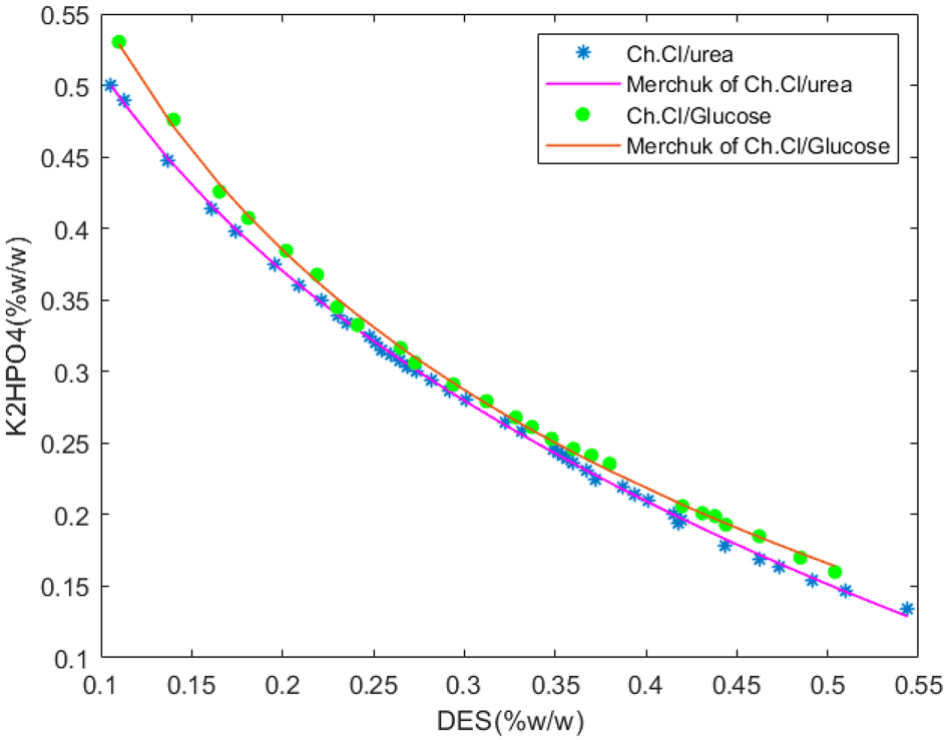
Binodal curve for both systems studied.

The cloud point approach at 25 °C and ambient pressure have been used to draw the binodal curve^24^. When selecting the salt to be used, two factors should be taken into account: first, that it has a strong ability to generate two phases, and second, that its pH range is good for *Haematococcus* microalgae. *H. pluvialis* was chosen for this investigation because dipotassium hydrogen phosphate salt has a potent ability to generate two phases and a pH range that is acceptable for microalgae (pH = 6–8)^19^. For both systems under study, the binodal curve is shown in Fig. 1.

The Merchuck equation was then applied to the binodal curve points that were obtained experimentally^9^.

1 $$Y=A.{\text{exp}}\left[\left(B.{X}^{0.5}\right)-(C.{X}^{3})\right]$$

According to the data from the binodal curve, X and Y in this equation are, respectively, the weight percentages of DES and dipotassium hydrogen phosphate, and coefficients A, B, and C are the equation's regulatory parameters, which are listed in Table 1.

**Table 1:** Parameters of the Merchuck equation and correlation coefficient for two aqueous two-phase systems.

Aqueous two-phase system	A	B	C	R^2^
(Ch. Cl/U) + K_2_HPO_4_ + H_2_O	106.6	− 0.232	2.494 × 10^−6^	0.9699
(Ch. Cl/G) + K_2_HPO_4_ + H_2_O	128.8	− 0.2679	1.24 × 10^−6^	0.8899

#### Determination of Tie-Lines (TLL)

Tie lines have been established using the gravimetric approach^25^. In this procedure, the concentrations previously found in the two-phase range were used to create the ternary combination (${{\text{K}}}_{2}{{\text{HPO}}}_{4}$ + H2O + DES). After thorough mixing, it was left at room temperature for 24 h to attain equilibrium. Once two phases had formed, each of the upper and lower phases was carefully separated using a syringe and weighed.

The four equations of four unknowns (${Y}_{T}$ and ${Y}_{B}$, ${X}_{T}$ and ${X}_{B}$) should be solved in order to ascertain the composition of the components in the upper and lower phases and, as a result, to draw the tie lines.

2 $${Y}_{T}=A.exp\left[\left(B.{X}_{T}^{0.5}\right)-(C.{X}_{T}^{3})\right]$$

3 $${Y}_{B}=A.exp\left[\left(B.{X}_{B}^{0.5}\right)-(C.{X}_{B}^{3})\right]$$

4 $${Y}_{T}=\frac{{Y}_{M}}{\propto }-\frac{1-\propto }{\propto }{Y}_{B}$$

5 $${X}_{T}=\frac{{X}_{M}}{\propto }-\frac{1-\propto }{\propto }{X}_{B}$$

The letters M, T, and B represent the feed mixture, upper phase, and lower phase, respectively. The α parameter also expresses the weight ratio of the upper phase to the feed mixture.

The length of the tie line (TLL) is one approach to describing the aqueous two-phase system (ATPS). TLL stands for the distance along the line that connects the high and low phases of a specified ATPS. TLL is the sum of the squares representing the DES and salt concentration differences between the top and bottom phases^26^.

6 $$TLL=\sqrt{{({Y}_{Top}-{Y}_{Bottom})}^{2}+{({X}_{Top}-{X}_{Bottom})}^{2}}$$

The tie lines are frequently straight; therefore, Eq. 7 can be used to get the slope of the tie line (STL). As a result, it makes it easier to build more tie lines.

7 $$STL=\frac{{Y}_{Top}-{Y}_{Bottom}}{{X}_{Top}-{X}_{Bottom}}$$

**Figure 2:**
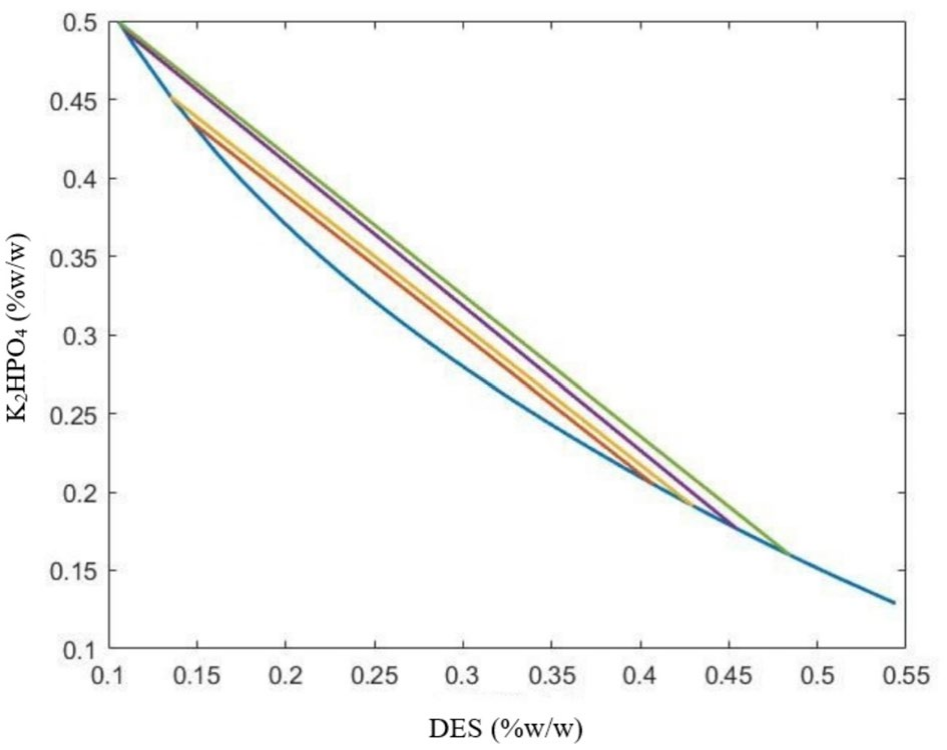
Tie lines of the aqueous two-phase system of choline chloride − urea + dipotassium hydrogen phosphate + water.

**Figure 3:**
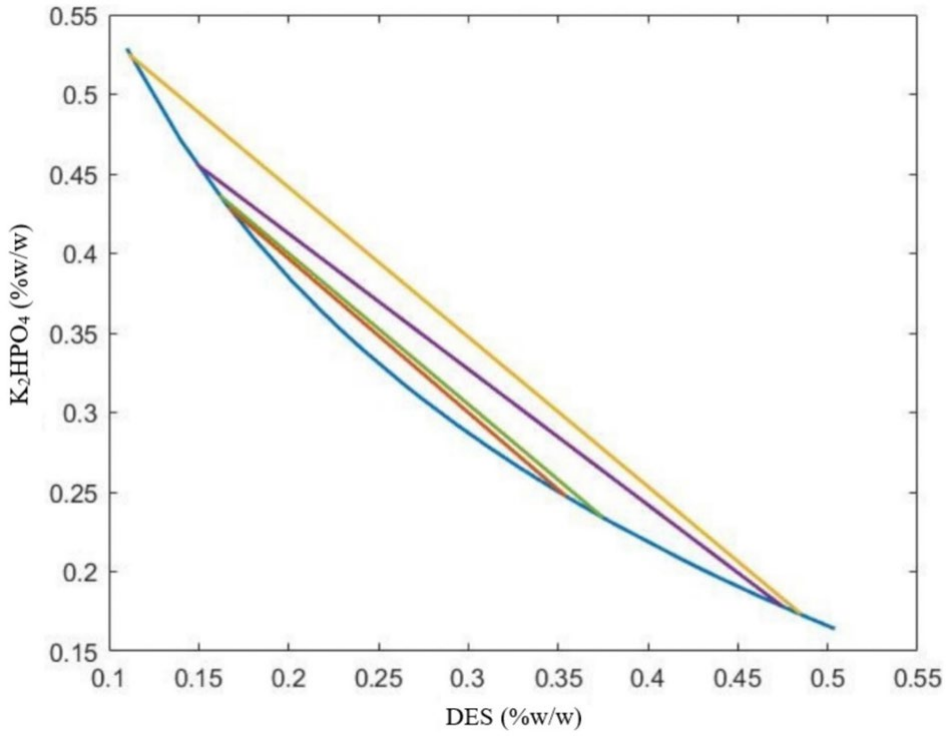
Tie lines of the aqueous two-phase system of choline chloride − glucose + dipotassium hydrogen phosphate + water.

Unknowns were used to determine the tie lines, which were then drawn using MATLAB software. The tie-line diagram for the dipotassium hydrogen phosphate salt and the choline chloride–urea system is shown in Fig. 2. The tie-line diagram for the dipotassium hydrogen phosphate salt and the choline chloride–glucose system is shown in Fig. 3.

The Atmer–Tobias relation compliance of the experimental data acquired for tie lines can be used to assess their thermodynamic consistency. A and B are the adjustment parameters in this equation^26–28^.

8 $$\mathit{ln}\left(\frac{100-{Y}_{T}}{{Y}_{T}}\right)=a+bln(\frac{100-{X}_{T}}{{X}_{T}})$$

Tables 2 and 3 show, for the choline chloride–urea and choline chloride–glucose systems, respectively, the weight percentage of the feed composition, upper phase and lower phase, tie line length, and slope.

**Table 2:** Characteristics of the two-phase aqueous system of choline chloride − urea + dipotassium hydrogen phosphate + water.

Feed number	Feed composition (percentage by weight)		Composition of the upper phase (percentage by weight)		Lower phase composition (weight percent)		STL	TLL
	DES	K_2_HPO_4_	DES	K_2_HPO_4_	DES	K_2_HPO_4_		
1	30 ± 1	30 ± 2	40.76 ± 0.5	20.47 ± 2	14.51 ± 0.6	43.72 ± 0.8	− 0.86 ± 0.03	35.07 ± 0.4
2	32 ± 0.5	30 ± 1	45.46 ± 0.7	17.64 ± 1	10.94 ± 0.4	49.33 ± 1	− 0.92 ± 0.01	46.86 ± 0.5
3	25 ± 0.8	35 ± 0.5	42.97 ± 0.9	19.11 ± 0.6	13.51 ± 0.7	45.16 ± 0.5	− 0.88 ± 0.05	39.32 ± 1
4	25 ± 1	37 ± 1	48.45 ± 1	15.97 ± 1	13.62 ± 1	49.89 ± 2	− 0.9 ± 0.02	50.83 ± 0.7

**Table 3:** Characteristics of the two-phase aqueous system of choline chloride − glucose + dipotassium hydrogen phosphate + water.

Feed number	Feed composition (percentage by weight)		Composition of the upper phase (percentage by weight)		Lower phase composition (weight percent)		STL	TLL
	DES	K_2_HPO_4_	DES	K_2_HPO_4_	DES	K_2_HPO_4_		
1	30 ± 2	30 ± 08	35.45 ± 2	24.73 ± 2	16.66 ± 0.8	42.9 ± 0.9	− 0.97 ± 0.01	26.13 ± 0.5
2	32 ± 1	30 ± 2	48.5 ± 2	17.35 ± 0.5	11.1 ± 0.4	52.52 ± 1	− 0.94 ± 0.01	51.34 ± 1
3	25 ± 0.7	37 ± 0.5	47.51 ± 0.6	17.79 ± 0.6	14.86 ± 1	45.63 ± 1	− 0.85 ± 0.03	42.91 ± 0.8
4	20 ± 1	40 ± 2	37.5 ± 1	23.4 ± 1	16.16 ± 0.8	43.65 ± 1	− 0.95 ± 0.02	29.4 ± 0.4

By comparing the experimental data with the Atmer–Tobias relations, the thermodynamic compatibility of the data obtained for the lines was examined. Table 4 provides the calculated values for the equation's constants and the correlation coefficient. The experimental data for tie lines are well-fitted by the Atmer–Tobias equation, as evidenced by the R^2^ values obtained for both systems.

**Table 4:** Values of constants and correlation coefficient for the correctness of tie lines with the help of Atmer–Tobias relationship.

Aqueous two-phase system	a	b	R^2^
(Ch. Cl/U) + K_2_HPO_4_ + H_2_O	1.719	− 0.9717	0.9999
(Ch. Cl/G) + K_2_HPO_4_ + H_2_O	1.612	− 0.8289	0.9999

### Liquid–liquid extraction process

#### Chlorophyll removal

Cells from *H. pluvialis* are first centrifuged for 20 min at 4,000 rpm. The following pellets were subjected to a solution of 5% (w/v) KOH and 30% (v/v) methanol at 70 °C for 10 min in order to remove total chlorophyll. The additional green material containing the extracted chlorophyll was thrown away following centrifugation at 4000 rpm for 10 min. The remaining microalgae cells were prepared for astaxanthin extraction and quantification by being rinsed twice with distilled water^29^.

#### Degradation of the cell wall of the microalgae *H. pluvialis* by DES

Five feeds with a specified percentage composition for each system in the biphasic area were weighed by a digital scale with an accuracy of 0.0001 g and put in 50-ml glass vials in accordance with the binodal curve that was derived for the two analyzed systems. This solution was used as a control solution. Since astaxanthin has a stable pH of 7.5, the sample's pH was measured using a pH meter, and depending on the type of system, its pH was changed by adding acid or base to bring it to 7.5. The initial pH of the deep eutectic solvent system containing choline chloride–urea was 9.5, which was adjusted to 7.5 by adding a few drops of 1 M hydrochloric acid. The initial pH of the deep eutectic solvent system containing choline chloride–glucose was 4, which was adjusted by adding a few drops of sodium. 1 M hydroxide was adjusted to 7.5. After removing the chlorophyll, the prepared oral solution was added to the microalgal cell residues to perform the pre-treatment process. The samples were treated for 1 h at 60°C on a magnetic stirrer. Then they were kept at laboratory temperature (25 ± 1°C) for 24 h to separate the phases. The upper phase, which is rich in deep eutectic solvent, was separated from the lower phase, which is rich in salt, and the destroyed cells that were located at the boundary of the two phases were separated using a syringe. After separating the destroyed cells, the cells were washed once with distilled water to perform the liquid–liquid extraction process to finally separate astaxanthin.

#### Extraction of astaxanthin by liquid–liquid method

To extract astaxanthin from cells pre-treated with a deep eutectic solvent, the Kuan et al. method was used^30^. The pre-treated cells were mixed with 15 ml of ammonium sulfate salt solution ${\left({NH}_{4}\right)}_{2}{SO}_{4}$ at 350 g/liter. Then 15 ml of 100% undiluted 2-propanol solution was added to the system. The flotation period was considered to be 15 min. Then, as shown in Figure S1, the upper and lower phases were carefully separated to estimate the distribution and percentage of astaxanthin extraction.

## Results and discussion

### Estimation of astaxanthin distribution

To obtain maximum light absorption by astaxanthin, 1 mg of 99.7% pure astaxanthin powder (HPLC) was dissolved in 10 ml of 99% pure 2-propanol. The standard characteristic absorption spectrum of astaxanthin was scanned in the range of 200–800 nm by a spectrophotometer, with maximum light absorption at 490 nm. Figure S2 shows the determination of the maximum absorption wavelength for astaxanthin by spectrophotometer.

Finding the standard curve is important before determining the astaxanthin concentration in phases. The standard curve can be established for this purpose by creating diluted solutions with various weight percentages of astaxanthin in the 2-propanol solvent, and a 99% pure 2-propanol solvent was used as a control^30^. The astaxanthin standard curve is depicted in Figure S3.

### Calculation of distribution coefficient, a volume ratio of phases, and recovery percentage

Equation 9 was used to determine the astaxanthin (${K}_{AS}$) distribution coefficient^31^.

9 $${K}_{As}=\frac{{AS}_{T}}{{AS}_{B}}$$

The concentration of astaxanthin is in the high and low phases, respectively, in the high relation (${AS}_{T}$) and (${AS}_{B}$).

The volume ratio of the phases (V_R_) was calculated using Eq. 10, in which (V_T_) and (V_B_) are the volumes of the upper and lower phases, respectively^30–32^.

10 $${V}_{R}=\frac{{V}_{T}}{{V}_{B}}$$

The extraction efficiency (E) of the upper phase is equal to the amount of biomolecule in the upper phase divided by the total biomolecule in the feed, which was calculated by Eq. 11^33^.

11 $${E}_{AS}=\frac{{K}_{As}\times {V}_{R}}{1+{K}_{As}\times {V}_{R}}$$

Table 5 lists the values for both systems distribution coefficients, volume ratios of the phases, and recovery percentages for each phase, along with the standard deviation (for three iterations of the experiment).

**Table 5:** V_R_, K_AS_ and E_AS_ parameter values for both systems.

Type of deep eutectic solvent	Feed composition (%)		V_R_	K_AS_	E_AS_ (%)
	DES	K_2_HPO_4_			
Choline chloride − urea	35	25	0.827 ± 0.02	305.79 ± 1.31	99.6 ± 0.2
	35	27	0.826 ± 0.01	320.58 ± 1.27	99.62 ± 0.08
	35	30	0.827 ± 0.02	341.58 ± 1.34	99.64 ± 0.05
	32	30	0.825 ± 0.005	317.78 ± 1.4	99.62 ± 0.05
	30	30	0.826 ± 0.01	291.05 ± 1.25	99.58 ± 0.03
Choline chloride − glucose	35	25	0.824 ± 0.004	235.06 ± 1.33	99.57 ± 0.1
	35	27	0.824 ± 0.006	246.64 ± 1.29	99.59 ± 0.09
	35	30	0.825 ± 0.005	274.25 ± 1.26	99.62 ± 0.03
	32	30	0.826 ± 0.01	269.37 ± 1.43	99.62 ± 0.04
	30	30	0.825 ± 0.01	253.59 ± 1.37	99.6 ± 0.02

### Factors affecting the distribution coefficient of astaxanthin

#### Influence of weight percentage of deep eutectic solvent

To investigate the effect of the weight percentage of DES, three feed groups with a constant weight percentage of dipotassium hydrogen phosphate salt (30%) and three different weight percentages of DES (30%, 32%, and 35%) were tested for both systems. With increasing DES concentration, the amount of cell wall degradation and, consequently, the distributivity coefficient increased. Studies have shown that ionic liquids and deep eutectic solvents have a very high capacity for cellulose dissolution and are weakened by hydroxyl groups, leading to hydrogen bond interactions between cellulose chains^14,33^. More specifically, the hydrogen bond breaks the various cellulose chains as part of the process of soluble cellulose in DES, which is an electron–electron acceptor (EDA) complex generated between the oxygen and hydrogen atoms of cellulose and the charged species of DES. This indicates that DESs have a greater capacity to break up cellulose's hydrogen bonds since they have strong enough H bonds of their own^34^. The capacity and potential for breakdown of the microalga cell wall therefore increased with increasing DES concentration as well as the concentration of electron acceptor and electron receptor sets.

#### Influence of weight percentage of dipotassium hydrogen phosphate

Three feed groups with a constant DES percentage (35%) and three different percentages of dipotassium hydrogen phosphate (25%, 27%, and 30%) were examined for both systems in order to examine the impact of the weight percentage of dipotassium hydrogen phosphate salt. The findings demonstrated that disruption of the microalgal cell wall improved with increasing dipotassium hydrogen phosphate concentration. As a result, more astaxanthin was available for the following liquid–liquid extraction process, and the distribution coefficient increased because the salt solution was disruptive as the first step of the osmotic shock act.

#### Temperature effect

Temperature is one of the influential factors that affect the physical and chemical behavior of DES and, as a result, the destruction of the cell wall of* Haematococcus*. To investigate the effect of temperature on the coefficient of distribution in the two feed systems by combining 35% deep eutectic solvent and 30% dipotassium hydrogen phosphate and after adding microalgae biomass to the feed for one hour on a magnetic stirrer-heat was stirred at different temperatures of 20, 30, 40, 50, and 60 °C, and then after the liquid–liquid extraction process, the dispersion coefficient was calculated. Table 6 shows the variation of the distribution coefficient with temperature. As can be seen, the temperature rise has further weakened the *H. pluvialis* microalgae cells cell walls. As a result, there is more astaxanthin accessible for extraction during the liquid–liquid extraction stage, which has raised the distribution coefficient. The saturation or degradation of fat components and astaxanthin isomers is typically brought on by excessive heat stress during physicochemical extraction methods. Reyes et al. (2014^35^) investigated the recovery of astaxanthin from *H. pluvialis* cyst biomass using supercritical ${CO}_{2}$ extraction. They recommended that the procedure temperature be kept below 50 °C to prevent astaxanthin's antioxidant activity from being noticeably diminished^35,36^. When pretreated with [Bmim]Cl and then extracted with methanol, Liu et al. (2018^34^) recently observed that the extractability of astaxanthin from *H. pluvialis* cyst cells increased proportionally with increasing temperature from 30 to 70 °C^34^. This discovery is in line with our most recent data, which shows optimal values for an upward trend in the 20–60°C temperature range.

**Table 6:** Distribution coefficient changes with temperature for the studied systems.

T (°C)	Ch. Cl/Urea	Ch. Cl/Glucose
	K_As_	K_As_
20	296.2 ± 6	237.37 ± 4
30	308.4 ± 5	246.64 ± 4
40	320.59 ± 8	259.53 ± 3
50	330.35 ± 4	255.91 ± 5
60	341.58 ± 5	274.25 ± 6

#### Effect of temperature on intermolecular interaction

Four different temperatures (20, 30, 40, 50, and 60 °C) were chosen to investigate the effect of temperature on the distribution of components. According to Eqs. 12–15, thermodynamic parameters can be determined^37,38^.

12 $$ln{K}_{As}=-\frac{{\Delta G}_{As}}{RT}$$

13 $$ln{K}_{As}=\frac{{\Delta S}_{As}}{R}-\frac{{\Delta H}_{As}}{RT}$$

14 $${\Delta G}_{As}={\Delta H}_{As}-T{\Delta S}_{As}$$

15 $$\upzeta =\frac{{\Delta H}_{As}}{{\Delta H}_{As}+T{\Delta S}_{As}}$$

The effect of temperature on the distribution coefficient and thermodynamic parameters at five different temperatures for choline chloride–urea and choline chloride–glucose systems are reported in Tables 7 and 8, respectively. The results show that increasing the temperature increases the distribution coefficient (K_As_). The process is a spontaneous and endothermic process because ∆H_As_ and ∆G_As_ are positive and negative, respectively, and ∆S_As_ shows the decrease or increase in the number of components distributed in the systems.

**Table 7:** Thermodynamic parameters for choline chloride–urea system.

T (**°**K)	K_As_	∆H_As_ (kj mol^−1^)	∆S_As_ (kj mol^−1^ K^−1^)	∆G_As_ (kj mol^−1^)	T*∆S_As_	ζ
293.15	296.2	2876.73	57.14	− 13,873.3	16,749.99	0.1466
303.15	308.4	2876.73	57.14	− 14,444.6	17,321.37	0.1424
313.15	320.59	2876.73	57.14	− 15,016	17,892.75	0.1385
323.15	330.35	2876.73	57.14	− 15,587.4	18,464.13	0.1348
333.15	341.58	2876.73	57.14	− 16,158.8	19,035.51	0.1313

**Table 8:** Thermodynamic parameters for the choline chloride–glucose system.

T (°K)	K_As_	∆H_As_ (kj mol^−1^)	∆S_As_ (kj mol^−1^ K^−1^)	∆G_As_ (kj mol^−1^)	T*∆S_As_	ζ
293.15	237.37	2632.212	54.44	− 13,325.4	15,957.64	0.1416
303.15	246.64	2632.212	54.44	− 13,869.8	16,502	0.1376
313.15	253.59	2632.212	54.44	− 14,414.1	17,046.35	0.1338
323.15	255.91	2632.212	54.44	− 14,958.5	17,590.7	0.1302
333.15	274.25	2632.212	54.44	− 15,502.8	18,135/05	0.1267

#### pH effect

**Figure 4:**
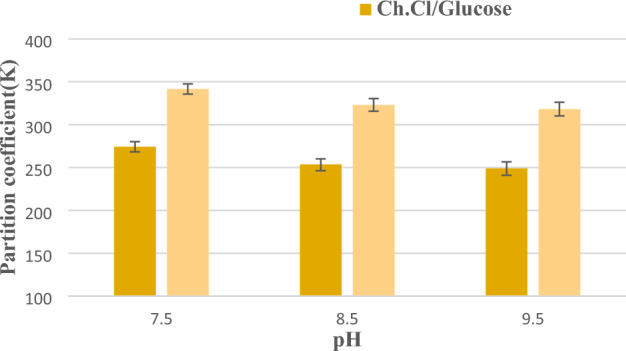
Distribution coefficient changes with pH for the studied systems.

One of the parameters that has the greatest effect on the extraction efficiency of most biomolecules, including astaxanthin, is pH. The graph of changes in distribution coefficient with pH for the studied systems is shown in Fig. 4. Changes in pH can affect the net charge of astaxanthin, the charge distribution on the surface of the microalgae, and the zeta potential, and severe pH can damage the structure of astaxanthin. Positively charged deep eutectic solvent cations can interact electrostatically with the negatively charged microalgae surface at the working pH. Therefore, time–load interactions can play an essential role in the extraction process. However, pH is not essential for improving extraction efficiency, possibly because pH only affects the surface charge of microalgae. Therefore, electrostatic interaction is not the main factor affecting astaxanthin extraction**.**

### Extraction mechanism

#### UV visible spectroscopy analysis

**Figure 5:**
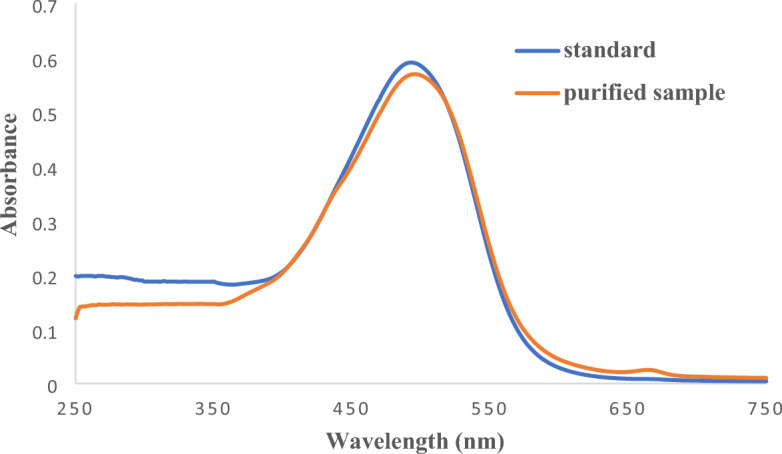
UV spectroscopy of standard astaxanthin solution in 2-propanol alcohol and extracted astaxanthin.

Visible spectroscopic analysis was performed to evaluate the structural stability of astaxanthin before and after extraction. Figure 5 shows the spectroscopy of a pure astaxanthin solution in 2-propanol alcohol and high phase after pre-treatment by DES and extraction with an astaxanthin-rich 2-propanol-ammonium disulfate 2-phase system. As can be seen, both have the same spectrum, and the maximum light absorption occurred at a wavelength of 490 nm. As a result, it can be said that astaxanthin has no reaction with any of the components of the system when its cell wall is destroyed by aqueous two-phase systems based on deep eutectic solvents and then liquid–liquid extraction with 2-propanol and ammonium disulfate salt. It will not be destroyed.

#### Fourier transform infrared spectroscopy (FTIR) analysis

**Figure 6:**
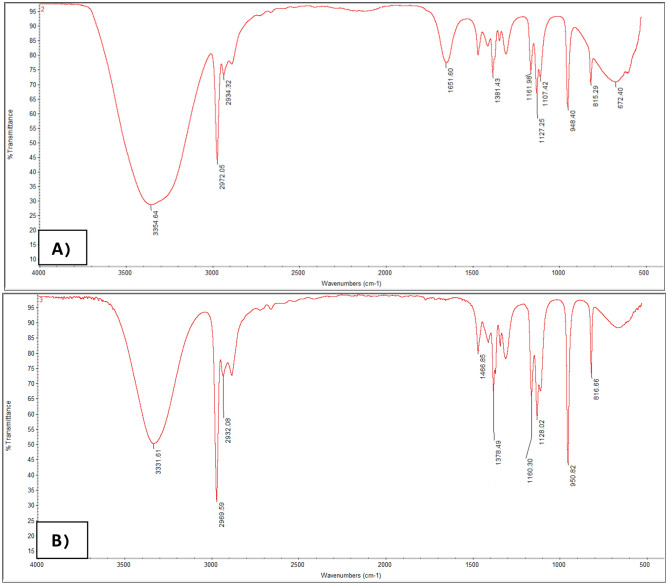
FTIR spectroscopic analysis A) Pure astaxanthin B) Extracted astaxanthin.

Factor groups are examined by FTIR spectroscopic analysis. In the present study, spectroscopy of pure astaxanthin and extracted astaxanthin were performed after cell wall destruction. The reason for this test is to investigate the chemical bonds and estimate the interactions in the extraction system, this test can be a confirmation of the stability of the structure of astaxanthin during the extraction process. Figure 6 shows the spectroscopy of (a) pure astaxanthin and (b) extracted astaxanthin, respectively. According to Fig. 6a pure astaxanthin has specific adsorption peaks in 3354 (O–H tensile bond), 2972 (C–H3 aliphatic tensile bond), 1651 (C = O ester tensile bond), 2002 (C = C bond) and 948 (C–H bond). Figure 6b, which is related to extracted astaxanthin, has all the main peaks of astaxanthin, and no additional peak is observed, so it can be concluded that the structure of astaxanthin has not changed and no new bond has been formed.

#### Microscopic observation and mechanism of cell wall disruption

**Figure 7:**
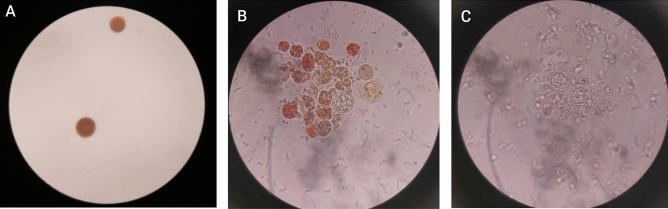
Microscopic images of *H. pluvialis* microalgal cell wall disorders (**A**) healthy *Haematococcus* cysts (**B**) ruptured cysts after pretreatment (**C**) colorless *Haematococcus* cysts after the astaxanthin extraction process.

The morphological alterations of *H. pluvialis* cyst cells were evaluated under a light microscope in order to comprehend the combined effects of the pre-treatment of a two-phase system based on a deep eutectic solvent and subsequent liquid–liquid extraction. Figure 7 illustrates these morphological changes. The intact cyst cells dramatically burst and discharged cytoplasmic components when the cells were treated with both methods. Also, red astaxanthin adhesive droplets were observed among the cell debris. In addition, after subsequent liquid–liquid extractions, cellular abnormalities increased. Some empty cells were visible. Notably, DES-based ATPS-treated cells have lost their cell wall integrity. In addition, after liquid–liquid extraction, all microalgal cells were almost colorless, indicating improved efficiency in the extraction of fat and astaxanthin. After therapy with DES-based aqueous two-phase systems, the microscopic characteristics of cell wall abnormalities were different from those found by Liu et al. (2018) and Desai et al. (2016). According to Liu et al. (2018), after pre-treatment with [Bmim] Cl, the cell surface of *H. pluvialis* exhibits numerous holes and becomes rough and wrinkled under light microscopy and scanning electron microscopy (SEM). It's interesting to note that Desai et al. (2016^39^) were unable to detect any appreciable alterations in the cell surface of *Haematococcus* cysts treated with [Bmim] DBP using light microscopy and SEM analysis. They suggested that subsequent recovery of astaxanthin by ethyl acetate solvent may be enhanced by the dissolution of the outer layer or penetration of IL into cells. These two reports suggest that the effect on the surface of *Haematococcus* cyst cells can be very different from that of IL^34,39^. It should also be noted that water content, extraction time, and temperature conditions can affect cell degradation performance. However, it can be concluded that the two systems tested in the present study can break the cell wall of *H. pluvialis* cysts effectively.

An adult *H. pluvialis* cystic cell with a high astaxanthin content has a resistant three-layer wall comprising approximately 16% of its dry cell weight. The cell wall consists of an outer sheath consisting of algae (a strong acetolysis-resistant aliphatic biopolymer), a very thick secondary layer below composed of cellulose and mannose with a homogeneous arrangement, and finally, inside a third wall consisting of cellulose and mannose, there is heterogeneity^37–40^. Hydrophilic imidazolium-based ionic liquids and deep eutectic solvents form the cellulose hydrogen bonding network in lignocellulosic and microalgal biomasses based on the mechanism of glycosidic bond hydrolysis through IL and DES (directly) and (indirectly) in the presence of hydroxy generated IL and DES. Oxygen and hydrogen atoms from OH groups in cellulose can form electron acceptor and donor complexes. The IL and DES anions combine to bind hydrogen atoms to cellulose by hydrogen bonding, while their cation interacts with oxygen atoms in cellulose through such bonding. In addition, IL and DES molecules can dissolve and separate water molecules and are further decomposed into hydronium and hydroxide ions. The resulting hydroxide anion can act as a nucleus on the carbon of the glucopyranoside unit. Through the synergistic effect of IL ions and hydroxides, the glycosidic bonds of cellulose can be effectively broken.

### Reuse of deep eutectic solvent

**Figure 8:**
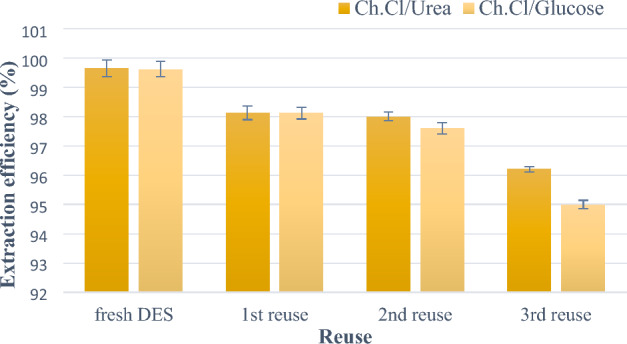
Performance of recycled DES.

The reuse of deep eutectic solvents is essential to reducing costs. For this purpose, reuse studies were performed using the same primary eutectic solvent for the pre-treatment of four new *H. pluvialis* cells. DES efficacy was tested by measuring the ability of DES to penetrate cells, resulting in the release of astaxanthin. The amount of astaxanthin that could be reused using DES was comparable to the control. DES can be used three times without any treatment or affecting the permeability performance. A decrease in permeability efficiency was observed when used for the fourth time for cell permeability. The deep eutectic solvent-rich layer was tested for the presence of pigment after each reuse, and it was observed that the amount of pigment in the DES phase after the three reuse cycles was negligible. The performance of recycled DES is shown in Fig. 8.

## Conclusion

In this study, new two-phase systems based on deep eutectic solvent systems No. 1 (Ch. Cl/Urea + ${{\text{K}}}_{2}{{\text{HPO}}}_{4}$+${H}_{2}O$) and No. 2 (Ch. Cl/Glucose + ${{\text{K}}}_{2}{{\text{HPO}}}_{4}$+${H}_{2}O$) were presented to investigate the degradation of the cell wall of *H. pluvialis*. Were. The results showed that the system containing a deep eutectic solvent (choline chloride–urea) had a better binodal curve and fuzzy separation ability. To investigate the cell wall degradation by the studied systems, five feeds with different combinations of percentages were considered for each system, and the pH of all systems was set at 7.5. Also, the effect of parameters such as temperature, pH, the weight percentage of the deep autistic solvent, and dipotassium dihydrogen phosphate salt on the distribution coefficient was investigated. The results showed that with increasing the weight percentage of deep eutectic solvent and dipotassium hydrogen phosphate salt, the distribution coefficient also increased. Increasing the temperature also has a positive effect on the process of cell wall degradation and increases the distribution coefficient, but it is important to note that high temperatures cause the degradation of lipids and astaxanthin. The extraction mechanism was investigated by UV, FTIR, and light microscopy analyses to ensure that the studied systems did not degrade astaxanthin. In the best operating conditions, the distribution coefficient and recovery percentage of astaxanthin for system number 1 were 341.58 and 99.64%, respectively, and for system number 2, they were 274.25 and 99.62%, respectively. Finally, after reviewing all the analyses and results, it can be claimed that both systems have a very high potential for degradation of the cell wall of the microalgae *H. pluvialis* and facilitate the next process, which is liquid–liquid extraction.

### Supplementary Information

Supplementary Figures.

## Data Availability

All data generated or analyzed during this study are included in this published article [and its supplementary information files].

## References

[1] Kim DY (2016). Cell-wall disruption and lipid/astaxanthin extraction from microalgae: Chlorella and Haematococcus. Bioresour. Technol..

[2] Chisti Y (2007). Biodiesel from microalgae. Biotechnol. Adv..

[3] Mularczyk M, Michalak I, Marycz K (2020). Astaxanthin and other nutrients from haematococcus pluvialis—Multifunctional applications. Mar. Drugs.

[4] Di Lena G, Casini I, Lucarini M, Lombardi-Boccia G (2019). Carotenoid profiling of five microalgae species from large-scale production. Food Res. Int..

[5] Wang F, Gao B, Wu M, Huang L, Zhang C (2019). A novel strategy for the hyper-production of astaxanthin from the newly isolated microalga Haematococcus pluvialis JNU35. Algal Res..

[6] Ma R (2018). Blue light enhances astaxanthin biosynthesis metabolism and extraction efficiency in Haematococcus pluvialis by inducing haematocyst germination. Algal Res..

[7] Sun H, Liu B, Lu X, Cheng KW, Chen F (2017). Staged cultivation enhances biomass accumulation in the green growth phase of Haematococcus pluvialis. Bioresour. Technol..

[8] Ahmed F, Li Y, Fanning K, Netzel M, Schenk PM (2015). Effect of drying, storage temperature and air exposure on astaxanthin stability from Haematococcus pluvialis. Food Res. Int..

[9] Gao J, Fang C, Lin Y, Nie F, Ji H, Liu S (2020). Enhanced extraction of astaxanthin using aqueous biphasic systems composed of ionic liquids and potassium phosphate. Food Chem..

[10] Pitacco W (2022). Extraction of astaxanthin from Haematococcus pluvialis with hydrophobic deep eutectic solvents based on oleic acid. Food Chem..

[11] Huang WC, Liu H, Sun W, Xue C, Mao X (2018). Effective Astaxanthin extraction from wet haematococcus pluvialis using switchable hydrophilicity solvents. ACS Sustain. Chem. Eng..

[12] Kim B, Youn Lee S, Lakshmi Narasimhan A, Kim S, Oh YK (2022). Cell disruption and astaxanthin extraction from Haematococcus pluvialis: Recent advances. Bioresour. Technol..

[13] Wilawan B (2023). Advancement of carotenogenesis of Astaxanthin from Haematococcus pluvialis: Recent insight and way forward. Mol. Biotechnol..

[14] Liu ZW, Yue Z, Zeng XA, Cheng JH, Aadil RM (2019). Ionic liquid as an effective solvent for cell wall deconstructing through astaxanthin extraction from Haematococcus pluvialis. Int. J. Food Sci. Technol..

[15] Paquin F, Rivnay J, Salleo A, Stingelin N, Silva C (2015). Multi-phase semicrystalline microstructures drive exciton dissociation in neat plastic semiconductors. J. Mater. Chem. C.

[16] Darani SF, Ahsaie FG, Pazuki G, Abdolrahimi S (2021). Aqueous two-phase systems based on thermo-separating copolymer for partitioning of doxorubicin. J. Mol. Liq..

[17] Ebrahimi A, Pazuki G, Mozaffarian M, Ahsaie FG, Abedini H (2023). Separation and purification of C-phycocyanin from spirulina platensis using aqueous two-phase systems based on triblock thermosensitive copolymers. Food Bioprocess Technol..

[18] P. Domínguez de María, Z. Maugeri, Ionic liquids in biotransformations: From proof-of-concept to emerging deep-eutectic-solvents. *Curr. Opin. Chem. Biol.*, **15**(2), 220–225, , 10.1016/j.cbpa.2010.11.008 (2011). 10.1016/j.cbpa.2010.11.008 21112808

[19] Lu W, Alam MA, Pan Y, Wu J, Wang Z, Yuan Z (2016). A new approach of microalgal biomass pretreatment using deep eutectic solvents for enhanced lipid recovery for biodiesel production. Bioresour. Technol..

[20] Wang Q, Wei N, Wang Y, Hou Y, Ren X, Wei Q (2020). Single-step purification of C-phycocyanin from Arthrospira platensis using aqueous two-phase system based on natural deep eutectic solvents. J. Appl. Phycol..

[21] Ding W (2019). Enhancing Haematococcus pluvialis biomass and Γ-aminobutyric acid accumulation by two-step cultivation and salt supplementation. Bioresour. Technol..

[22] Li F (2019). Differences between motile and nonmotile cells of haematococcus pluvialis in the production of astaxanthin at different light intensities. Mar. Drugs.

[23] Liyanaarachchi VC, Nishshanka GKSH, Premaratne RGMM, Ariyadasa TU, Nimarshana PHV, Malik A (2020). *Astaxanthin* accumulation in the green microalga *Haematococcus pluvialis*: Effect of initial phosphate concentration and stepwise/continuous light stress. Biotechnol. Rep..

[24] Sintra TE (2021). Sequential recovery of C-phycocyanin and chlorophylls from Anabaena cylindrica. Sep. Purif. Technol..

[25] de Araujo Sampaio D (2016). Assessment of sodium salt anions (SO42- and NO3-) influence on caffeine partitioning in polyethylene glycol and 1-butyl-3-methylimidazolium tetrafluoroborate based ATPS. J. Solution Chem..

[26] Nandini KE, Rastogi NK (2011). Liquid–Liquid extraction of lipase using aqueous two-phase system. Food Bioprocess. Technol..

[27] Murari GF (2015). Phase diagrams of aqueous two-phase systems formed by polyethylene glycol+ammonium sulfate+water: Equilibrium data and thermodynamic modeling. Fluid Phase Equilib..

[28] Taghavivand M, Pazuki G (2014). A new biocompatible gentle aqueous biphasic system in cefalexin partitioning containing nonionic Tween 20 surfactant and three organic/inorganic different salts. Fluid Phase Equilib..

[29] Sarada R, Vidhyavathi R, Usha D, Ravishankar GA (2006). An efficient method for extraction of astaxanthin from green alga Haematococcus pluvialis. J. Agric. Food Chem..

[30] Khoo KS, Chew KW, Ooi CW, Ong HC, Ling TC, Show PL (2019). Extraction of natural astaxanthin from Haematococcus pluvialis using liquid biphasic flotation system. Bioresour. Technol..

[31] Chong KY, Stefanova R, Zhang J, Brooks MSL (2020). Extraction of bioactive compounds from Haskap Leaves (Lonicera caerulea) using salt/ethanol aqueous two-phase flotation. Food Bioprocess Technol..

[32] Zhu Y (2021). Simultaneous promotion of photosynthesis and astaxanthin accumulation during two stages of Haematococcus pluvialis with ammonium ferric citrate. Sci. Total Environ..

[33] Phong WN (2017). Proteins recovery from wet microalgae using liquid biphasic flotation (LBF). Bioresour. Technol..

[34] Liu ZW, Zeng XA, Cheng JH, Liu DB, Aadil RM (2018). The efficiency and comparison of novel techniques for cell wall disruption in astaxanthin extraction from Haematococcus pluvialis. Int. J. Food Sci. Technol..

[35] Reyes FA, Mendiola JA, Ibañez E, Del Valle JM (2014). Astaxanthin extraction from Haematococcus pluvialis using CO 2-expanded ethanol. J. Supercrit. Fluids.

[36] Choi SA (2019). Astaxanthin extraction from Haematococcus pluvialis using CO2-expanded ethanol. The Journal of Supercritical Fluids.

[37] Ahsaie FG, Pazuki G (2021). Separation of phenyl acetic acid and 6-aminopenicillanic acid applying aqueous two-phase systems based on copolymers and salts. Sci. Rep..

[38] Ghasemzadeh B, Shahriari S, Pazuki G (2019). Efficient separation of curcumin using tetra butyl phosphonium bromide/carbohydrates (sorbitol, fructose) aqueous two-phase system. Fluid Phase Equilib..

[39] Desai RK, Streefland M, Wijffels RH, Eppink MHM (2016). Novel astaxanthin extraction from Haematococcus pluvialis using cell permeabilising ionic liquids. Green Chem..

[40] R. K. Desai, *Ionic liquid pre-treatment of microalgae and extraction of biomolecules* (2016).

