# Supplementary material for: Extraction and separation of astaxanthin with the help of pre-treatment of Haematococcus pluvialis microalgae biomass using aqueous two-phase systems based on deep eutectic solvents
Source: Sci Rep. 2024 Mar 5;14:5420. doi: 10.1038/s41598-024-55630-4 (PMC10914728; doi:10.1038/s41598-024-55630-4)
Supplement: Supplementary file 1 — Supplementary Figures. [file 41598_2024_55630_MOESM1_ESM.docx]

**Supplementary Information**

**Extraction and separation of astaxanthin with the help of pre-treatment of Haematococcus pluvialis microalgae biomass using aqueous two-phase systems based on deep eutectic solvents**

**Neda Nemani ^a^, Seyed Mohsen Dehnavi ^b^, Gholamreza Pazuki ^a, *^**

^a^ Department of Chemical Engineering, Amirkabir University of Technology (Tehran Polytechnic), Tehran, Iran

^b^ Department of Cell and Molecular Biology, Faculty of Life Science and Biotechnology, Shahid Beheshti University, P.O. Box 1983969411, Tehran, Iran.

* Corresponding author: [ghpazuki@aut.ac.ir](mailto:ghpazuki@aut.ac.ir) ,Tel: +98-021-64543159, Fax: +98-021-66405847

Table S1. Materials used to make Bold's Basal medium (BBM) culture medium

| Mg/L | material |
| --- | --- |
| 250 | NaNO_3_ |
| 75 | MgSO_4_.7H_2_O |
| 75 | K_2_HPO_4_ |
| 175 | KH_2_PO_4_ |
| 25 | NaCl |
| 25 | CaCL_2_.2H_2_O |
| 4.98 | FeSO_4_.7H_2_O |
| 50 | EDTA |
| 31 | KOH |
| 0.49 | CO(NO_3_)_2_.6H_2_O |
| 8.82 | ZnSO_4_.4H_2_O |
| 1.44 | MnCL_2_.4H_2_O |
| 0.71 | MoO_3_ |
| 1.57 | CuSO_4_.5H_2_O |
| 11.42 | H_3_BO_3_ |

Table S2. The results of Karl Fischer titrator analysis

| Sample water content (%) | System |
| --- | --- |
| 1.428±0.43 | Ch. Cl/Urea |
| 1.798±0.21 | Ch. Cl/Glucose |


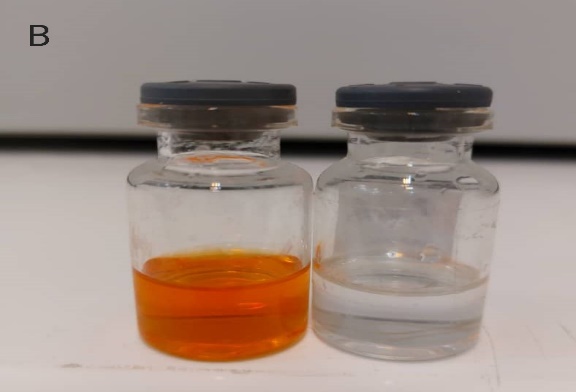

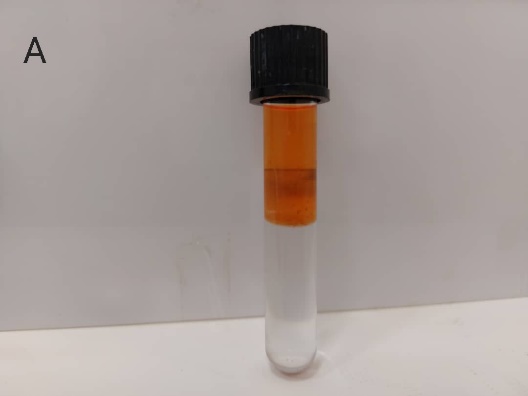


Figure S1. The liquid-liquid extraction process of astaxanthin a) after 24 hours of standing in the laboratory environment b) after separating each of the phases by syringe

Table S3. Binodal curve data for both systems in terms of total weight percentage for components

| DES (Ch. Cl/Glucose) + K_2_HPO_4_ | | | DES (Ch. Cl/Urea) + K_2_HPO_4_ | | |
| --- | --- | --- | --- | --- | --- |
| W_water_ (%) | W_K2HPO4_ (%) | W_DES_ (%) | W_water_ (%) | W_K2HPO4_ (%) | W_DES_ (%) |
| 33.6 | 16 | 50.4 | 32.2 | 13.4 | 54.4 |
| 34.5 | 17 | 48.5 | 34.86 | 14.2 | 50.94 |
| 35.27 | 18.5 | 46.23 | 35.69 | 15.16 | 49.15 |
| 35.3 | 19.3 | 44.4 | 36.64 | 16.05 | 47.31 |
| 36.3 | 19.9 | 43.8 | 37.02 | 16.76 | 46.22 |
| 35.8 | 20.1 | 43.1 | 37.85 | 17.83 | 44.32 |
| 37.4 | 20.6 | 42 | 38.84 | 19.36 | 41.8 |
| 38.44 | 23.56 | 38 | 38.47 | 19.59 | 41.94 |
| 38.86 | 24.14 | 37 | 38.48 | 20 | 41.52 |
| 39.3 | 24.6 | 36.1 | 38.77 | 21.11 | 40.12 |
| 39.9 | 25.3 | 34.8 | 39.02 | 21.64 | 39.34 |
| 40.15 | 26.13 | 33.72 | 39.15 | 22.15 | 38.7 |
| 40.36 | 26.8 | 32.84 | 39.94 | 22.83 | 37.23 |
| 40.84 | 27.93 | 31.23 | 40.01 | 23.26 | 36.73 |
| 41.5 | 29.1 | 29.4 | 40.15 | 23.88 | 35.97 |
| 42.08 | 30.62 | 27.3 | 40.15 | 24.16 | 35.69 |
| 41.85 | 31.65 | 26.5 | 40.18 | 24.41 | 35.41 |
| 42.63 | 33.25 | 24.12 | 40.12 | 24.71 | 35.17 |
| 42.5 | 34.5 | 23 | 40.16 | 24.96 | 34.88 |
| 41.34 | 36.76 | 21.9 | 41.02 | 25.85 | 33.13 |
| 41.36 | 38.44 | 20.2 | 41.36 | 26.43 | 32.21 |
| 41.15 | 40.73 | 18.12 | 41.91 | 27.99 | 30.1 |
| 40.88 | 42.58 | 16.54 | 42.07 | 28.71 | 29.22 |
| 38.35 | 47.6 | 14.05 | 42.35 | 29.44 | 28.21 |
| 35.97 | 53.03 | 11 | 42.58 | 30.02 | 27.4 |
|  |  |  | 42.73 | 30.38 | 26.89 |
|  |  |  | 42.78 | 30.79 | 26.43 |
|  |  |  | 42.86 | 31.15 | 25.99 |
|  |  |  | 43.08 | 31.46 | 25.46 |
|  |  |  | 42.87 | 32 | 25.13 |
|  |  |  | 42.71 | 32.48 | 24.81 |
|  |  |  | 43.01 | 33.41 | 23.58 |
|  |  |  | 43 | 33.95 | 23.05 |
|  |  |  | 42.88 | 35.01 | 22.11 |
|  |  |  | 43.13 | 35.98 | 20.89 |
|  |  |  | 42.92 | 37.49 | 19.59 |
|  |  |  | 42.79 | 39.76 | 17.45 |
|  |  |  | 42.53 | 41.36 | 16.11 |
|  |  |  | 42.08 | 44.25 | 13.67 |
|  |  |  | 39.7 | 49 | 11.3 |
|  |  |  | 39.42 | 50 | 10.58 |

Figure S2. Determination of the maximum absorption wavelength for astaxanthin by spectrophotometer

Figure S3. Standard curve of astaxanthin in 2-propanol
